# Associations between national tuberculosis program budgets and tuberculosis outcomes: an ecological study

**Published:** 2012-07-09

**Authors:** Will Chapple, Alan Roy Katz, Dongmei Li

**Affiliations:** 1 Department of Public Health Sciences, John A. Burns School of Medicine, University of Hawai'i at Manoa, USA

**Keywords:** National tuberculosis program, budget, tuberculosis

## Abstract

**Introduction:**

The objective of this study is to explore the associations between national tuberculosis program (NTP) budget allocation and tuberculosis related outcomes in the World Health Organization's 22 high burden countries from 2007–2009.

**Methods:**

This ecological study used mixed effects and generalized estimating equation models to identify independent associations between NTP budget allocations and various tuberculosis related outcomes. Models were adjusted for a number of independent variables previously noted to be associated with tuberculosis incidence.

**Results:**

Increasing the percent of the NTP budget for advocacy, communication and social mobilization was associated with an increase in the case detection rate. Increasing TB-HIV funding was associated with an increase in HIV testing among TB patients. Increasing the percent of the population covered by the Directly Observed Therapy (DOT) program was associated with an increase in drug susceptibility testing. Laboratory funding was positively associated with tuberculosis notification. Increasing the budgets for first line drugs, management and multi-drug resistant tuberculosis (MDR-TB) was associated with a decrease in smear positive deaths.

**Conclusion:**

Effective TB control is a complex and multifaceted challenge. This study revealed a number of budget allocation related factors associated with improved TB outcome parameters. If confirmed with future longitudinal studies, these findings could help guide NTP managers with allocation decisions.

## Introduction

The World Health Organization (WHO) estimates that a third of the world's population is infected with *Mycobacterium tuberculosis* [1]. TB was the eighth leading cause of death in the world in 2008 [2]. Approximately 80% of all infections occur in the WHO's 22 high burden countries [3].

Sputum smear positive cases are a top priority of the WHO and national tuberculosis programs (NTP). Many control measures focus on smear positive cases since they are the most infectious [4]. The WHO defines the case detection rate (CDR) as the number of smear positive cases reported divided by the number of estimated smear positive cases for that year [5]. Case notification is the process of reporting TB cases to the WHO [5]. Early case detection serves a dual purpose of identifying individuals who may require isolation until treatment is initiated and ensuring TB treatment is delivered before the disease progresses [6].

The growing realization of the importance of community involvement in the Directly Observed Therapy (DOT) program and in public health campaigns has lead to national funding for programs supporting advocacy, communication and social mobilization (ACSM) and community based TB care (CTBC). ACSM promotes advocating for national governments to strengthen TB control programs, changing attitudes and behaviors that work against TB control, and mobilization of stakeholders and communities to participate in TB prevention [7]. Community based TB care (CTBC) also mobilizes the community to prevent the spread of TB [8, 9].

There have been no published studies that we are aware of which attempt to explore the impact of national TB program budgetary allocations on TB outcomes in those countries with the highest TB burden. An earlier ecological study by Dye et al. assessed the relationship between annual change in TB incidence and various determinants, other than NTP budgetary allocations, in 134 countries from 1997-2006 [10].

The objective of this study is to explore the associations between NTP budget allocation and TB related outcomes in the WHO's 22 high burden countries from 2007-2009. The purpose of exposing these associations is to identify optimal allocation strategies and guide future studies towards problem areas that, if addressed, can improve the global response to TB.

## Methods

Mixed effects and generalized estimating equation (GEE) models were used to test the independent association between NTP budget allocation related variables and TB outcomes. The key independent variables included percent of NTP budget for first line drugs, administration, laboratory equipment and supplies, ACSM/CTBC, TB-HIV, MDR-TB, staff, and DOT. To explore population level factors, additional independent variables included NTP budget per capita, NTP budget per gross domestic product, and percent of the population covered by DOT (Table 1).

**Table 1:** Independent variables related to tuberculosis outcomes in 22 high burden countries*

Outcomes	Source	Independent Variables	Sources
TB Incidence per 100,000	14	% NTP Budget for Management and Administration	8,9,19
Retreatments per TB Incidence	14 Derived	% NTP Budget for Staff	8,9,19
TB Case Detection Rate (%)	14	% NTP Budget for First-Line Drugs	8,9,19
Smear Positive TB Deaths per Smear Positive TB Incidence	14 Derived	% NTP Budget for Laboratory Equipment and Supplies	8,9,19
Relapse TB cases per TB Incidence	14 Derived	%NTP Budget for MDR-TB	8,9,19
Defaulted Retreatment TB Cases per Retreatment TB Cases	14 Derived	% NTP Budget for TB-HIV	8,9,19
Incidence of Extrapulmonary TB per TB Incidence	14 Derived	% NTP Budget for ACSM/CTBC	8,9,19
TB Prevalence per 100,000	14	% NTP Budget for DOT Program	8,9,19
TB Notification per TB Incidence	14 Derived	NTP Budget/Capita	8,9,14,19 Derived
Drug Susceptibility Tests per TB Incidence	14 Derived	NTP Budget/GDP	8,9,14,19 Derived
HIV Testing Among TB Patients per TB Incidence	14 Derived	Percent of the Population Covered by DOT Program	3
TB Mortality per 100,000	14	-	-
**Covariates from Dye *et al.* [10]**		**Source**	
Human Development Index		15	
Corruption Perception Index		16	
GDP/Capita		17	
% of Population With Income <$2/day		17	
Percentage of Foreign-Born TB Patients		17	
Diabetes Prevalence age 20-79		17	
% of Population Undernourished		17	
% of Female Population That Smokes		17	
HIV Prevalence		14	
HIV Prevalence in TB Patients		14	
% of Population With Improved Water Source (Urban)		17	
% of Population With Improved Water Source (Rural)		17	
% of Population With Improved Sanitation (Urban)		17	
% of Population With Improved Sanitation (Rural)		17	
% of Population Using Solid Fuel		18	
Total Health Expenditure/Capita		17	
Government Health Expenditure /Capita		17	
Total Health Expenditure/GDP		17	
Physicians/100,000		17	
Under-5 Mortalities		17	
Annual Change in Under-5 Mortalities		10	
Expenditure on TB Control/Capita		8,9,19	
New Smear Positive Cases Detected		14	

* Afghanistan, Bangladesh, Brazil, Cambodia, China, D.R. Congo, Ethiopia, India, Indonesia, Kenya, Mozambique, Myanmar, Nigeria, Pakistan, Philippines, Russia, South Africa, Tanzania, Thailand, Uganda, Vietnam, Zimbabwe

Covariates included as potential confounding variables were taken from Dye et al.'s ecological study [10], and included all variables that demonstrated a Pearson's correlation greater than 0.20 for annual change in TB incidence using univariate regression analysis. These included economic factors, population make-up, population health factors, health services, and factors related to a country's TB control program (Table 1).

Dependent/outcome measurements for this analysis included TB incidence, TB specific mortality rate, ratio of retreatment to incidence, CDR, the ratio of smear positive deaths to smear positive incidence, ratio of relapse to incidence, ratio of defaulted retreatment cases to retreatment cases, proportion of new cases which were extrapulmonary, TB prevalence, ratio of notification to incidence, proportion of new cases that underwent susceptibility testing, and the proportion of incident cases who were tested for HIV (Table 1). Outcome measurements were chosen based on applicable influence to TB control and also the availability of the data. Mixed effects model was used for continuous normally distributed TB outcomes such as TB incidence, CDR, ratio of notification to incidence, and proportion of new cases that underwent susceptibility testing. The GEE model was used when the normality assumption was invalid for the TB outcomes such as the proportion of incident cases who were tested for HIV and the ratio of smear positive deaths to smear positive incidence. Each TB outcome measurement was tested one-by-one using a multivariate model with all aforementioned independent variables and covariates.

Data was included for each of the 22 high burden countries from 2007-2009. If data was not available for a particular year, the most recent data was used. If there was no data available after the year 2000, then no data was entered into the model. The study was exempt from IRB review as it was a secondary data analysis using aggregated existing data without identifiers.

### Statistical Analysis

Pearson correlations were examined among independent variables to avoid potential multicollinearity problem in the model fitting. If two (or more) variables were found to be highly correlated (r > 0.70) only one was included in the model. Increasing the total sample size and diversity of variables were considered when choosing the highly correlated variable to be included. The variables that were excluded due to high correlation were annual change in GDP, % of the population with income less than $1 purchasing power parity (PPP), income inequality, % of the population less than 15 years old, % of the population in urban areas, % of adult males that smoke, expenditure on TB control per patient, new TB cases and smear positive cases detected, treatment success in DOT cohort, and new TB and smear positive treatment success rates. Mixed effects models were fitted to the TB related outcomes in the WHO's 22 high burden countries from 2007-2009 using the proc mixed procedure in SAS 9.1.3 (SAS Institute, Cary, NC) with repeated statements to account for the correlations of TB related outcomes from the same country across years. The Akaike information criteria (AIC) was used to assess the model fit. Residual diagnostics were conducted to check the multivariate normality assumption of the mixed effects model. If the assumption was violated, either a log transformation of the TB related outcome was used or a GEE model was used instead to fit the TB related outcomes. The GEE models can also take the correlation of TB related outcomes from the same country across multiple years into account and were fitted to the TB related outcomes using the proc genmod procedure in SAS 9.1.3. The Quasilikelihood under the Independence model Criterion (QIC) statistic (analogous to AIC [11, 12]) was used to examine the goodness fit of the GEE models (Table 2).

**Table 2:** Significant results relating key independent variables to TB outcomes

Outcome variable	Significant key independent variable	Effect on outcome variable after a 1% increase of the key independent variable	95% CI	P-value	Fit
TB Incidence per 100,000	% of NTP budget for MDR-TB	Increase of 1 case per 100,000	0.99–1.00 case per 100,000	0.032	AIC−27.0
TB Case detection rate (%)	% of NTP budget for ACSM/CTBC	0.27% increase	0.07%–0.47%	0.02	AIC 166.6
Smear Positive TB deaths per smear positive TB cases	% of NTP budget for First-Line Drugs	0.1% decrease	0.02%–0.2%	0.02	QIC 2.4112
	% of NTP budget for Management and Administration	0.13% decrease	0.09%–0.17%	<0.0001	
	% of NTP budget for MDR-TB	0.05% decrease	0.01%–0.09%	0.007	
	% of NTP budget for DOT	0.11% increase	0.04%–0.17%	0.0002	
	NTP budget/GDP (std dev = 0.0013)	0.9% increase	0.55%–1.2%	<0.0001	
Notification per TB incidence	% of NTP budget for Laboratory supplies and equipment	0.18% increase	0.012%–0.34%	0.05	AIC−15.7
Drug susceptibility testing among TB patients per TB incidence	% of the population covered by DOT	114% increase	95%–130%	0.01	AIC 75.3
HIV testing among TB patients per TB incidence	% of NTP budget for TB-HIV	1.13% increase	0.6%–1.6%	<0.0001	QIC 44.0707
	NTP budget/GDP (std dev = 0.0013)	7% decrease	1.7%–12%	0.009	

AIC: Akaike information criteria; QIC: Quasilikelihood under the Independence model Criterion

For both mixed effects models and GEE models, the purposeful model building procedure proposed by Hosmer and Lemeshow [13] was used to first select covariates independently associated with the TB related outcomes in univariate analyses with the criteria of P-values ≤ 0.20 for entering the model. Multivariate analyses were then conducted to select significant predictor variables for each of the TB related outcomes based on both the Wald chi-square tests and likelihood ratio tests with the criteria of P-values ≤ 0.05. The confounding variables that made the changes of the estimated coefficients of other independent variables in the models greater than 20% were kept in the model to adjust the confounding effects on the TB related outcomes. The estimated coefficients and their 95% confidence intervals were used to measure the magnitude of the association between NTP budget allocation related variables and TB related outcomes.

## Results

In most cases, only 18 or 19 countries could be included in the models due to missing data. All key independent and outcome variables were from 2007-2009, while some covariates were from 2006.

Increasing the percent of the NTP budget for MDR-TB by 1% was associated with an increase in the estimated TB incidence of 1 case per 100,000 population. An increase in the NTP budget for ACSM/CTBC of 1% was associated with an increase of 0.27% in the TB case detection rate.

An increase in the NTP budget for first line drugs by 1% was associated with a decrease of 0.1% in the ratio of smear positive TB deaths per smear positive TB cases. When the percent of the NTP budget for management and administration increased by 1% there was an associated decrease of 0.13% in this ratio, and when the percent of the NTP budget for MDR-TB increased by 1% there was an associated decrease of 0.05% in this ratio. When the percent of the NTP budget for the DOT program increased by 1% there was an associated increase in this ratio of 0.11% and an increase of 0.9% when the NTP budget per GDP increased by 0.13%.

When the percent of the NTP budget for laboratory supplies and equipment increased by 1%, there was an associated increase in the TB notification per TB incidence ratio of 0.18%. When the percent of the population covered by the DOT program increased by 1%, there was an associated increase in the ratio of drug susceptibility tests (DST) per TB incidence of 114%.

As the percent of the NTP budget for TB-HIV increased by 1%, the ratio of HIV testing among TB patients per TB incidence increased by 1.13%. This ratio was associated with a decrease of 7% when the ratio of NTP budget per GDP increased by 0.13% (Table 2).

No significant associations were found between the previously listed independent variables and the following outcome variables: ratio of retreatment to TB incidence, TB specific mortality rate, ratio of defaulted retreatment cases to retreatment cases, ratio of TB relapse to TB incidence, proportion of incident TB cases that are extrapulmonary, and TB prevalence.

## Discussion

It is important to note that disease prevention and epidemiology is a multifactorial issue. With budgetary constraints and overall NTP resource limitations this study hopes to provide data that can be used by NTP program administrators to help make difficult reallocation decisions.

An important finding of this study was the association between increased NTP budget allocation for ACSM/CTBC and an increase in the CDR. Improving the amount of government and community participation and knowledge about TB and its risk factors through ACSM/CTBC would facilitate the integration of the NTP and DOT programs in the communities in which they operate and thus raise the CDR. If these findings are confirmed, then a country that wishes to improve its CDR of TB could focus its budget accordingly.

Another important finding was that an increase in the percent of the population covered by the DOT program was associated with a dramatic increase in the rate of drug susceptibility testing. Increasing population coverage by the DOT program could be an important strategy when trying to battle MDR-TB. However, this may also reflect an association between increased DOT coverage and more complete reporting of DST.

India has the highest number of TB incident cases in the world. If the results of this study were applied to India in 2009, then 5,400 more TB cases would have been detected after a 1% increase in the NTP budget for ACSM/CTBC. There would have been 1,722 more DST and 22,600 more HIV tests done for TB patients after a 1% increase in the percent of the population covered by DOT and a 1% increase in the NTP budget for TB-HIV respectively. Finally, 1,748 smear positive TB deaths would have been prevented after a 1% increase in the NTP budgets for first line drugs, management and administration, and MDR-TB [3].

Some of the results are expected and further validate this analysis. The results show that increasing TB-HIV funding is associated with improved HIV testing rates among TB patients per incidence, and increased laboratory funding is associated with improved notification per incidence. Other results may be explained by the non-time span nature of this study. For example, the finding that an increase in the MDR-TB budget (funding for laboratory diagnosis and drug treatment for MDR-TB) was associated with an increase in the TB incidence per 100,000 population may reflect that countries with high TB incidence also tend to have high rates of MDR-TB and have allocated their NTP budgets appropriately. However, it is also possible that funding MDR-TB can take away focus and money from primary TB prevention hence leading to an adverse outcome.

The finding of a direct association between NTP budget per GDP with high rates of smear positive deaths per smear positive cases may reflect the fact that countries with greater smear positive death rates are allocating more NTP funding per GDP to address this issue. While the non-time span, cross-sectional nature of this study disallows temporal conclusions, it does not seem plausible that this is an inverse relationship: that increased NTP funding per GDP would cause an increase in smear positive death rates. The association between DOT funding increase and increased smear positive deaths may reflect an appropriate response to an increasing rate of smear positive deaths by increasing DOT funding. Conversely, this result could indicate that a focus on DOT without the equal inclusion of TB detection and DST may actually lead to an increase in smear positive deaths.

The ratio of smear positive deaths per smear positive cases was the outcome associated with and possibly influenced by the largest number of independent variables. This was perhaps one of the most important findings uncovered in this analysis. Increased budgetary allocations for front line drugs, management and administration, and addressing MDR-TB all were related to a significant decrease in this measurement. This highlights the many factors associated with smear positive deaths, and is a further reminder of the complex and multifaceted challenge posed by TB control. TB specific mortality rates are estimated by the WHO and are not as accurate as the smear positive death rates [14]. This may explain the discrepancy between the results for smear positive death rates and TB specific mortality.

While each dependent variable studied is an important TB-related outcome, it is not unexpected that variability in budget allocations would lead to variability in the outcome measures. The key independent variables studied reflect the diverse strategic approaches currently employed by NTPs for TB control. The NTP budgetary “pie” is composed of many pieces ranging from laboratory support, to therapeutic costs, to support for community advocacy and social mobilization. An increase in the overall NTP budgets of high burden TB countries would be desirable, but this may be an unrealistic goal especially in the current global economic environment. If TB outcomes can be positively influenced by NTP budgetary reallocations, a more realistic approach to TB control may be undertaken.

### Strengths and Limitations

The strength of this study is its ability to account for a wide array of variables, countries and years. It provides an overview of previously undetected associations between NTP budgets and operations with a variety of TB outcomes. This study used percentage of NTP budget allocations to categorize key independent variables rather than focusing on gross monetary expenditures or budget percentage increases in order to better capture the effects of TB budgetary allocations independent of budget size.

A limitation of the study is the ecological study design. Individual level conclusions cannot necessarily be drawn from aggregate data. However, the stated goal of this study was to explore the association between NTP budget allocations and TB related outcomes in order to help guide future individual level studies in directions previously unforeseen.

Missing data, the assumption that some data would not change from 2006-2009, the possibility of human error in the primary data collection and analysis, and reporting and estimation error in the primary databases are also limitations of this study. Use of CDR as an outcome indicator is also problematic in that it relies on estimated smear positive cases in its calculation and as such is prone to errors. The WHO is currently revising its strategy for the use of the smear positive CDR [3]. TB incidence measurements also rely on predictive models and are prone to error [9] and many of the outcome variables used TB incidence in the denominator. The fact that only the 22 high burden countries were included is a limitation. However, this is because the WHO only provided published data on NTP budget allocations for the 22 high burden countries from 2007-2009.

In addition, the covariates included in the model as potential confounders were variables associated with annual change in TB incidence taken from an ecological study. While many of these variables have been shown to be significantly associated in individual level studies, some were not. Furthermore, these covariates were in most cases only significant to a specific WHO region.

There are aspects of NTP budgetary interactions with TB outcomes that would be very useful to explore but could not be assessed in this study. Specifically, stratification of the results by country, WHO region, and HIV burden were not possible in this study because we focused only on high TB burden countries and the resulting sample size would be too small to have enough power to detect any significant associations between NTP budget allocation and TB outcomes. Future WHO datasets that include more than 3 years of reported data from the high burden countries or data from all countries beyond the high TB burden countries would yield important results and should be considered in future studies.

## Conclusion

It is recommended that the findings from this study be confirmed with individual level studies, such as case-control and cohort studies. It is also recommended that these findings be replicated using data from other countries in the WHO database to assess for consistency and to see if these results are generalizable beyond the high burden countries.
